# Supplementary material for: Steps dominate gas evasion from a mountain headwater stream
Source: Nat Commun. 2022 Dec 17;13:7803. doi: 10.1038/s41467-022-35552-3 (PMC9759591; doi:10.1038/s41467-022-35552-3)
Supplement: Supplementary file 1 — Supplementary Information [file 41467_2022_35552_MOESM1_ESM.pdf]

Steps dominate gas evasion from a mountain headwater stream - Supplementary Information

Gianluca Botter, Anna Carozzani, Paolo Peruzzo, Nicola Durighetto

April 2022

1 Supplementary Methods, Figures and Tables

1.1 Physics-based numerical simulation of gas evasion in a local step.

Flow discontinuity induced by local steps entails a marked heterogeneity in the hydrodynamic field, which is also mirrored by energy dissipation and gas evasion. Supplementary Figure 1 shows an example of the spatial patterns of turbulent kinetic energy dissipation rate, ε, calculated for an idealized step of 25 cm through a detailed $3D$ numerical simulation. We reproduced the hydrodynamics of a step in a 0.3 m wide rectangular channel for a steady flow rate of 2.0 l/s using the commercial software *Flow3D Hydro 2022R1* (Flow Science Inc., Santa Fe, NM, USA). To suitably discretize the numerical domain and reproduce the dynamics of the free surface, the software applied the fractional area/volume (FAVOR) and the volume-of-fluid (VOF) methods[1] on a grid of approximately 7.5 million structured orthogonal cells. The turbulent flow regime was simulated adopting the Reynolds-Averaged Navier-Stokes (RANS) scheme by resolving the turbulence at the sub-grid scale with the Re-Normalisation Group (RNG) $e_k - \varepsilon$ model (where e_k denotes the turbulent kinetic energy). The model also estimated the ensuing patterns of gas transfer rate, assuming a non linear dependence of k on ε (i.e. $k \propto \varepsilon^{0.25}$). According to our numerical simulation, the turbulent dissipation of energy was very high close to the plunging jet but it decreased by several orders of magnitude few centimeters downstream of the plunging jet - thereby resulting in a super-linear reduction of the local rate of gas exchange at the water-air interface. Consequently, as we increase the value of the length over which the process is averaged L - which in real-world experiments might correspond to the distance between the upstream and downstream recording positions of gas concentration - we also get a dramatic decrease of the mean value of $k(L)$. The simulation is only functional to illustrate the issue of scale-dependence of k in local steps (when the spatial patterns of ε and k are complex and typically unknown).

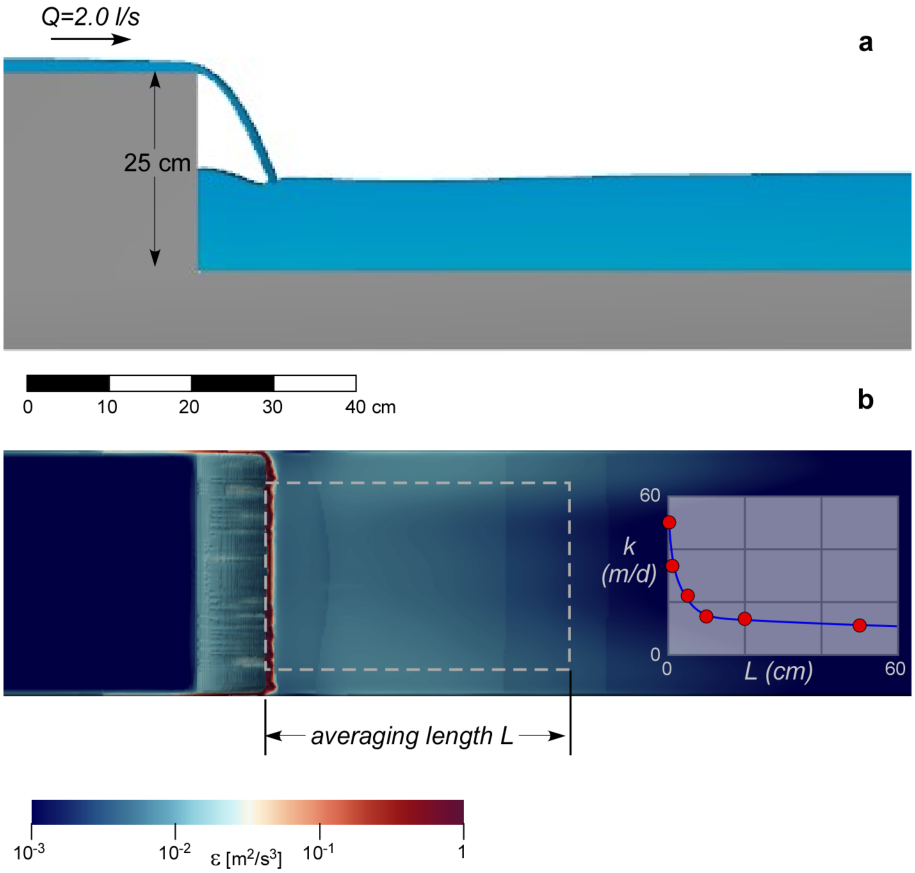

Supplementary Fig. 1: Numerical simulation of the hydrodynamics in a step. (a) lateral view of the free surface and step geometry. (b) color map of the kinetic turbulent dissipation rate, ε, computed at the free surface. In the inset, values of k as a function of the averaging length, L, where the mean turbulent dissipation rate, $\bar{\varepsilon}$, is estimated (grey dotted line). The gas transfer velocity is estimated as $k = 0.4\, Sc^{-0.5}(\nu\, \bar{\varepsilon})^{0.25}$ (Moog and Jirka[2]), where ν is the water kinematic viscosity and Sc is the Schmidt number here assumed equal to 600, i.e., $k = k_{600}$.

1.2 Damping factor aggregation across composite systems

Eq. (1) of the main text describes the evasion of a gas while it is transported downstream under steady state conditions, thereby providing a basis to characterize the spatial patterns of gas concentration along a reach under the assumptions listed in the main text. In particular, in this paper a reach was decomposed into sequences of steps and segments as shown in Fig. 2 and Supplementary Fig. 2. For the sake of illustration, here we calculate the damping factors and the dominance ratio of an ideal reach characterized by 3 segments with different slope interlaced by 2 steps (Supplementary Figure 2). Applying Eq. (1), the analytical expressions for the concentration at the downstream section of each reach element (segment or step), are the following:

$$C_2 = C_a + [C_1 - C_a] \exp[-f_{c_1}] \,, \tag{1}$$

$$C_3 = C_a + [C_2 - C_a] \exp[-f_{s_1}] \,, \tag{2}$$

$$C_4 = C_a + [C_3 - C_a] \exp[-f_{c_2}] \,, \tag{3}$$

$$C_5 = C_a + [C_4 - C_a] \exp[-f_{s_2}] \,, \tag{4}$$

$$C_6 = C_a + [C_5 - C_a] \exp[-f_{c_3}] \,. \tag{5}$$

Using the product of powers property, the concentration at the outlet of the reach portion (i.e. Section 6 of Supplementary Fig. 2) can be expressed in terms of the damping factors of all the 5 reach elements as:

$$C_6 = C_a + [C_1 - C_a] \exp[-(f_{c_1} + f_{s_1} + f_{c_2} + f_{s_2} + f_{c_3})] \,. \tag{6}$$

Grouping f of steps and segments and rearranging the terms inside the round brackets at the r.h.s. of the above equation, the downstream concentration C_6 can be expressed as:

$$\begin{aligned} C_6 &= C_a + [C_1 - C_a] \exp[-f_c - f_s] \\ &= C_a + [C_1 - C_a] \exp[-f_c(1 + r)] \,, \end{aligned} \tag{7}$$

where $f_c = f_{c_1} + f_{c_2}$ and $f_s = f_{s_1} + f_{s_2} + f_{s_3}$ are the reach-scale damping factors for the segments and the steps. The above equation shows that: i) the damping factors of different segments or steps are additive and commutative; ii) the relative contribution to the outgassing provided by the steps as compared to that provided by the segments is quantified by the step dominance ratio, $r = f_s/f_c$ (i.e. if $r = 1$ then the contribution of the steps and that of the segments to the total outgassing is the same, as discussed in the main text).

1.3 Discharge measurements

The volumetric discharge, Q [$\mathrm{L^3T^{-1}}$], which was estimated through the time needed to fill a graduated tank:

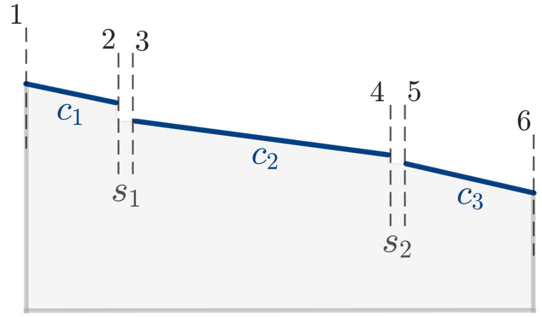

Supplementary Fig. 2: Schematization of a portion of stream reach decomposed into segments, c_i, and steps, s_i. Dashed lines defines upstream and downstream sections associated to each element.

clearpage

$$Q = \frac{V_0}{T_f} \,, \tag{8}$$

where V_0 [L^3] is the water volume collected in the tank during the filling time T_f [T^{-1}]. Volumetric discharge measurements, reported in Supplementary Table 1, were performed in the upstream (Supplementary Figure 9**a**) and downstream sections (Supplementary Figure 9**c**) of the representative segment, ℓ_r. Other point measurements were performed in corresponding of the steps using the same method (and in one case the method of the tracer dilution).

1.4 Travel time measurements and estimation of the relevant hydraulic properties in the reference segment

The water travel time in the reference segment, τ [T], was estimated as the time elapsed from the injection of the tracer in the upstream section to the peak time when the centroid of the plume is observed at the outlet of the segment. A solution of water, salt (50 mg/l) and a red dye were added in the upstream section and a multi-parameter sonde, YSI EXO2, was used to monitor the temporal dynamics of the specific conductivity, spCOND [S/L] in the downstream section of the representative segment. Travel time measurements are reported in Supplementary Table 1 where the mean values of 3 consecutive tests are indicated. Travel time measurements were performed before and after the streambed covering operations. These measurements were thus used to quantify possible variations of the velocity field due to PVC film on the streambed. The data show a clear power law relationship between travel time and discharge with different sets of model parameters for the undisturbed and streambed-covered conditions (Supplementary Figure 4). For low flows, in covered streambed condition travel time decreased with respect to natural conditions and, therefore, the PVC film increased the flow velocity. The opposite trend was observed for high flows.

Overall, the data indicate that small variations of the travel time with respect to the natural conditions were observed in the experiments carried out with the PVC film above the streambed. This in turn suggests that the hydrodynamic conditions of the water flow over the plastic film were somewhat similar to those observed in natural setting, in spite the lower hydraulic resistance implied by the film.

The hydraulic geometry relationships proposed by Leopold and Maddock were analyzed in covered streambed conditions (Supplementary Figure 5). Mean widths (W), mean water depths (H) and mean velocities (u_{cov}) were found to scale with the discharge (Q) as follows:

$$u_{cov} = 0.928\ Q^{\,0.298}\ , \tag{9}$$

$$W = 8.250\ Q^{\,0.540}\ , \tag{10}$$

$$H = 0.138\ Q^{\,0.170}\ . \tag{11}$$

The measurements carried out during our experiments in the representative segment were thus in line with scaling relationships proposed in literature. Overall, our data indicated that there was a limited impact of the film on the water travel times, suggesting that the presence of the plastic film did not generate a significant change of the roughness of the riverbed and the velocity field. This was likely due to the fact that gravels and stones were located over the film before letting the water flow back into the river, which helped us to reproduce near-natural conditions.

Supplementary Table 1: Measured volumetric discharges (Q), travel times in natural (τ_{nat}) and covered (τ_{cov}) streambed conditions, longitudinal mean velocity (u_{cov}), mean width (W), mean water depth (H) in the reference segment.

Date	Q $[l/s]$	τ_{nat} $[s]$	τ_{cov} $[s]$	u_{cov} $[m/s]$	W $[m]$	H $[m]$
10-Sep-2021	0.19	207	180	0.072	0.080	0.032
30-Sep-2021	0.32	163				
28-Oct-2021	0.35	160				
21-Sep-2021	0.37	157	148	0.088	0.120	0.035
02-Sep-2021	0.48					
20-Oct-2021	0.73	123	120	0.108	0.160	0.042
11-Oct-2021	1.87	81				
22-Jul-2021	2.00					
7-Oct-2021	2.11	77	88	0.148	0.300	0.048
14-Jul-2021	3.20					

a
b
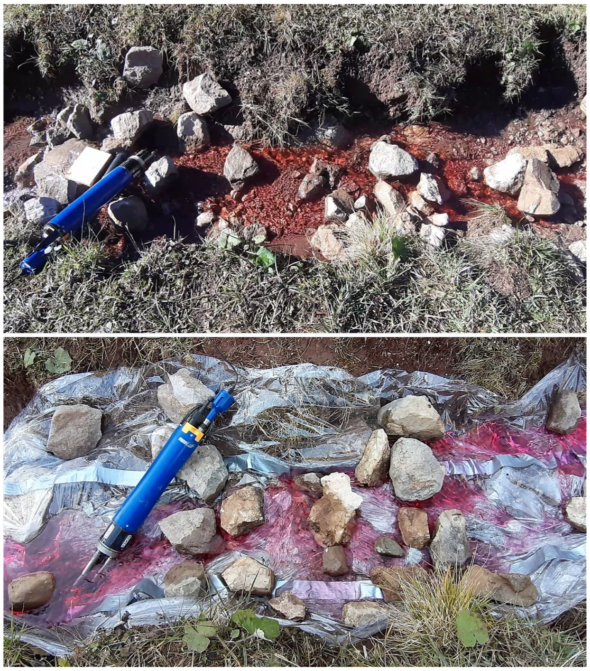

Supplementary Fig. 3: Travel time measurements by salt tracer addition with a red dye, using the YSI EXO2 sonde at the outlet section of the representative segment. On (**a**) the streambed was in natural condition (i.e. uncovered) while on (**b**) the PVC film covered the streambed.

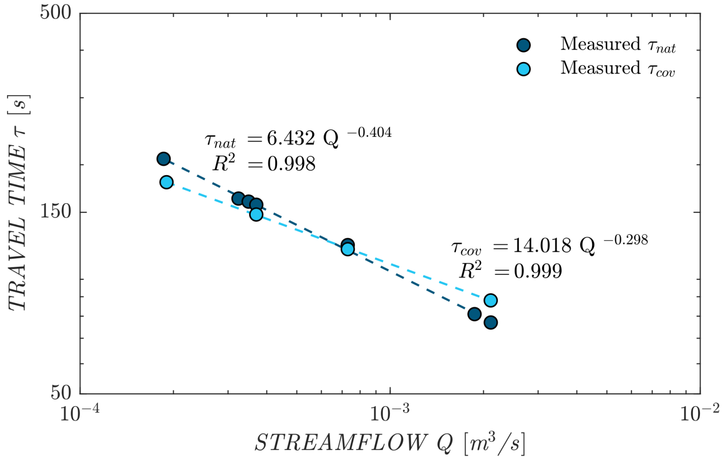

Supplementary Fig. 4: Log-log plot of measured travel times and discharges in uncovered (blue) and covered streambed (light blue) conditions with power law fitting and relative R-squared values.

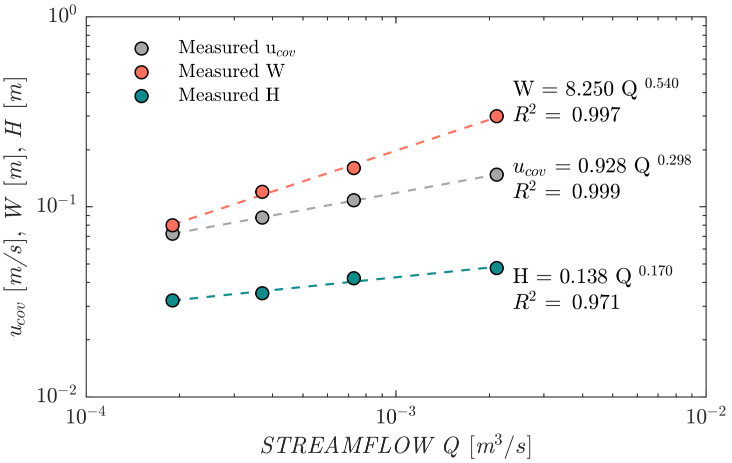

Supplementary Fig. 5: Log-log plot of measured discharges and mean stream velocities, mean widths and water depths in covered streambed condition with power law fitting and relative R-squared values.

1.5 Estimating f_{s_i} and f_{c_r}

1.5.1 General setup

To quantify the function $f_{s_i}(\Delta h_i)$, upstream and downstream gas concentrations in correspondence of the steps are required. This goal was reached by considering steps with different drop heights (i.e. gap of water surface elevation in vertical falls) and measuring water CO_2 concentrations right before and after the falling jet. In all cases, CO_2 measurements were performed by excluding the influence of ecosystem metabolism. A first group of measurements was performed by creating artificial steps above the covered stream segment, progressively adding 100 mm diameter PVC pipes to the intake located at the upstream section (Supplementary Figures 6 and 7**c**,**d**) or in other points along the focus reach (Figure 1**c** of the main text, and Supplementary Figure 8). The concentration upstream of each step was measured by inserting, at the end of the pipe, a PVC tee pipe fitting, which was used to place the probe into the water flow. The concentration immediately downstream of the falling jet was then evaluated by placing the sonde right below the falling jet, above the covered streambed. A second group of measurements pertained to natural steps along the tributary (Supplementary Figure 7**f** and Figure 1**d** of the main text). This was done to compare differences and analogies between natural steps and the artificial steps created through the pipe. In this case, to avoid internal production of CO_2, two different techniques were used: i) the streambed was manually scoured prior to the experiment and the biofilm was carefully removed; ii) the streambed was covered using a protection film in correspondence of the ramp, the step and the pool (Supplementary Figure 7**e**).

The damping factor of the segment, f_{c_r}, was estimated through CO_2 concentrations collected in the upstream and downstream sections of the 13 m representative segment (Figure 1**b** of the main text, Supplementary Figure 7**a**,**b**). In this case, to avoid internal production of CO_2, the streambed was covered using a plastic film as described in the main text. The major stages of the streambed-covering operations were the following: i) the diversion of the water through pipes to expose the stream bed to the air (Supplementary Figure 9**a**); ii) the wrapping of the dry bed with the plastic film and re-positioning of small rocks and boulders above it to recreate natural-like flow conditions (Supplementary Figure 9**b**); iii) finally, water was let flowing back over the covered streambed (Supplementary Figure 9**c**). These operations are better described in the following. First of all, the water in the upstream reach section was forced into a pipe of 100 mm diameter which served as a collector to divert the flow and exposed the streambed to the air. Pipes were added to divert the water on the hydrographic right side. While the water was diverted, the covering operation of the dry streambed was carried out using a transparent low density polyethylene stretch film for packaging. This stretch wrap was prepared in advance by joining three coils 500 mm high with waterproof tape, in order to have a sufficient width for wrapping the streambed and the lateral sides. The

small rocks and gravel material, previously removed from the streambed, were then relocated over the plastic film to maintain the hydraulic regime and the roughness of the reach segment. Lastly, the water was allowed to flow back over the covered streambed.

a b c

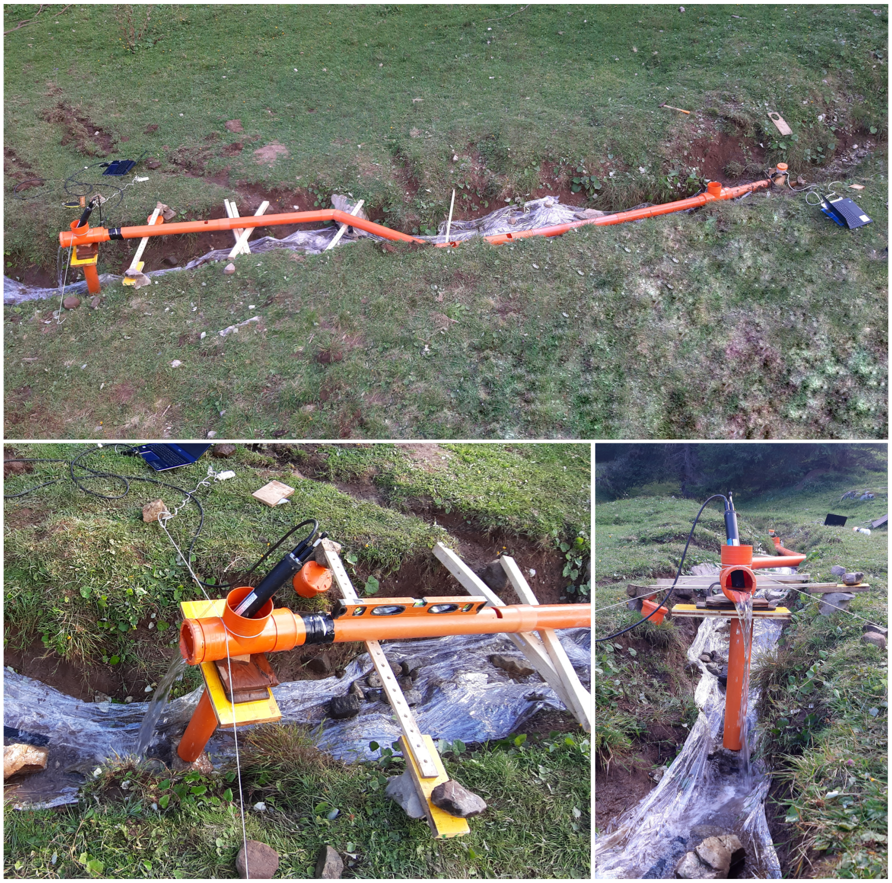

Supplementary Fig. 6: Simulated step created above the covered stream segment progressively adding 100 *mm* diameter PVC pipes to the intake located at the upstream section. Experiment performed on September 2021. a) Overview of the whole pipe set-up. b) Close-up view of the outlet. c) Front view of the outlet.

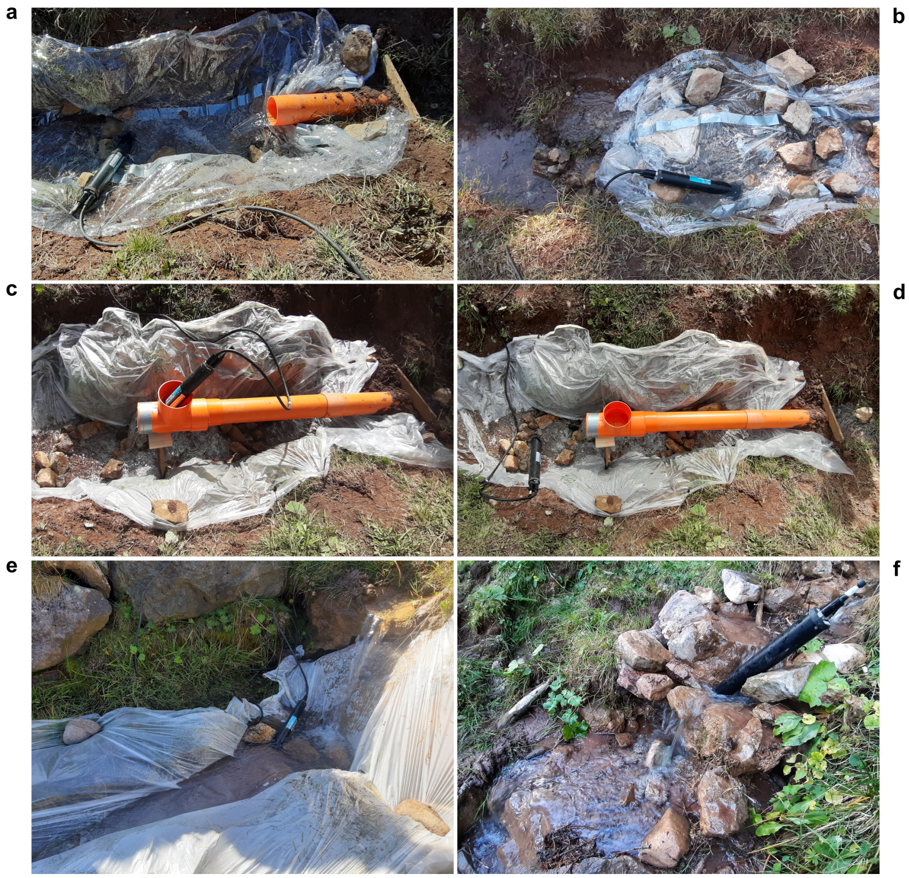

Supplementary Fig. 7: Experimental configurations. On (**a**) and (**b**) CO_2 concentration measurements in the upstream and downstream sections of the covered representative segment. On (**c**) and (**d**) the data collection for an artificial step 27 *cm* high, setting up by drain addition and using a pvc tee pipe-fitting to measure the upstream concentration. On (**e**) and (**f**) natural steps of drop height equal 58 and 20 *cm* respectively, in covered/uncovered streambed conditions.

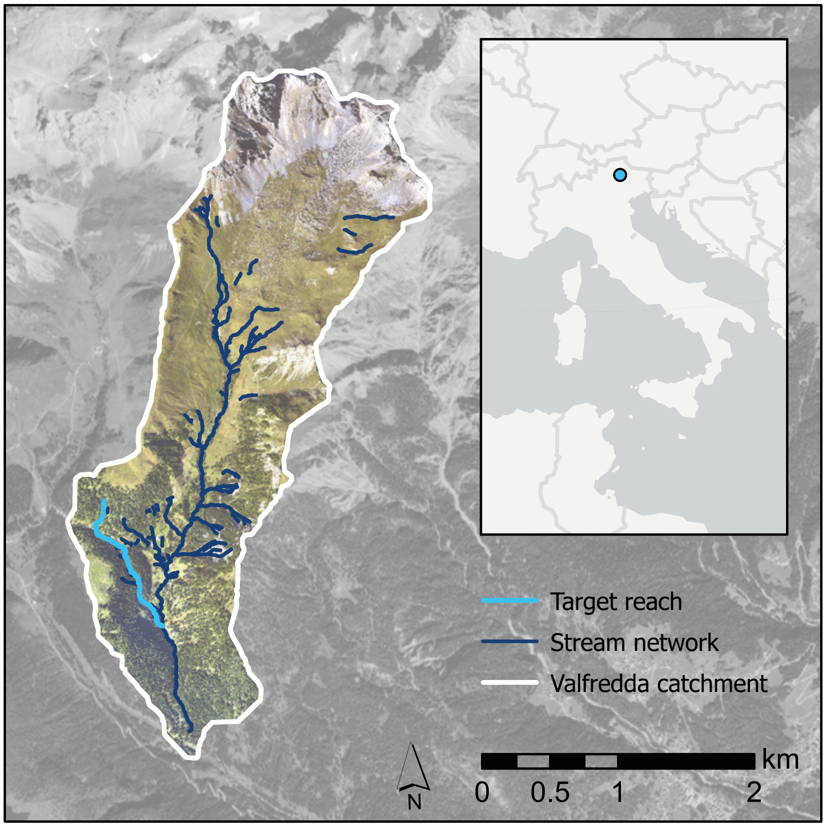

Supplementary Fig. 8: Study reach within the Valfredda creek and its contributing catchment. The location of the site in Italy is also shown (46°23'25" North and 11°50'18" East in the WGS84 coordinated system). Sources: Esri, DigitalGlobe, GeoEye, i-cubed, USDA FSA, USGS, AEX, Getmapping, Aerogrid, IGN, IGP, swisstopo, and the GIS User Community.

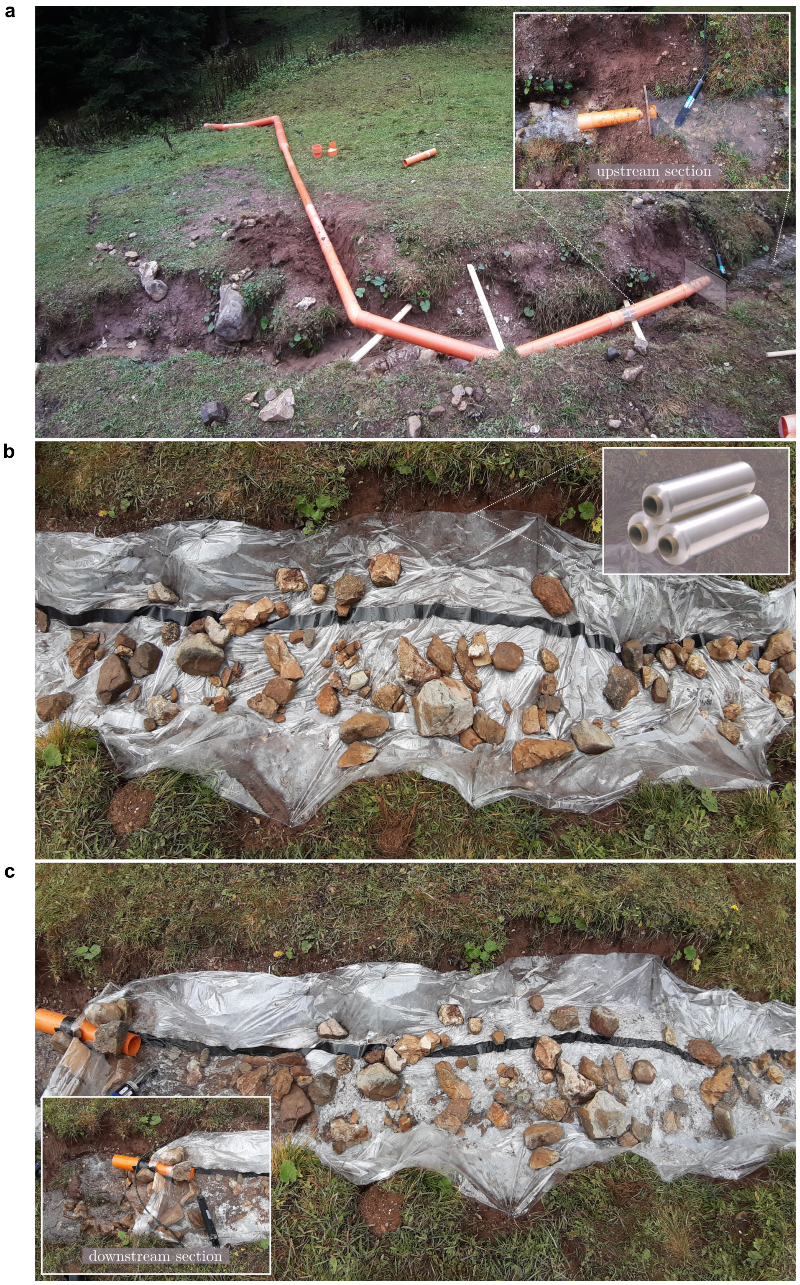

Supplementary Fig. 9: The 3 main stages of the covering operation performed in the representative segment: on (**a**) water diversion - photo taken on the hydrographic left side - with a plan view of the upstream section on the right top; on (**b**) wrapping with the PVC film and repositioning of small rocks and boulders; on (**c**) flowing back of the water over the covered streambed and view of the downstream section.

1.5.2 CO_2 concentration measurements

Carbon dioxide concentrations were collected by the MiniCO_2™ Submersible pCO_2 Sensor of the ProOceanus Systems Incorporated (PSI). The instrument is fitted with an equilibrator composed of a gas permeable membrane and an internal detection loop with a non-dispersive infrared detector (NDIR). In the tube, CO_2 gas molecules absorb the specific band so that the optical filter is reached by all remaining wavelengths and the detector "reads" the amount of residual frequencies not absorbed by gas molecules. The accuracy of the instrument is $\pm$ 2% and the water temperature range supported by the probe goes from -2° to $50\,°C$. During field measurements the instrument was set to take a measure every 4 seconds. The duration of any individual deployment to get a proper estimate of the local CO_2 concentration is regulated by different factors. An overly long immersion of the instrument in water is not recommended due to temporal variability of water CO_2 concentrations during the day. Moreover, the upstream and downstream data collection should be as synchronous as possible, which implies that the deployment in a given location should be as short as possible. On the other hand, the equilibrium concentration was reached in not less than 15 mins, even though the equilibration time was impacted by specific field conditions such as temperature, concentration differences, turbulence etc. High-frequency fluctuations in the signal of the MiniCO_2™ sensor were observed in field and laboratory applications. Signals impacted by random noises usually have a bell shaped probability density function (pdf). The mean of the pdf lies at the center of the distribution, and represents an unbiased estimate of the true mean based on all of the measured data. The standard deviation defines the width of the distribution, i.e. the extent of concentration temporal variations within a single deployment. Based on all the above considerations, for each location we measured the average values of dissolved CO_2 concentrations in pre-identified "plateaus" of the observed time series, with a minimum plateau duration of 10 mins. These temporal averages were considered as representative of the equilibrium carbon dioxide concentration in water and were used for the estimation of the damping factors. Even tough the frequency distribution of measured CO_2 concentrations could be used to estimate the uncertainty in the calculated damping factors and CO_2 fluxes, we decided to avoid this type of analysis as the focus of the paper does not revolve around this type of measurement uncertainty. Nonetheless, this procedure allowed a robust experimental identification of local CO_2 concentrations and damping factors, eliminating the effect of noise in the observed records.

1.5.3 CO_2 concentrations, CO_2 fluxes and damping factors for the reference segment

Carbon dioxide concentrations were collected from September to November 2021 in the upstream and downstream sections of representative segment. The ensuing time series of CO_2 concentrations are shown in Supplementary Figure 10,

jointly with the corresponding pdfs.

Estimated damping factors in the representative segment, f_{c_r}, were derived from measured upstream/downstream concentrations according to Eq. (8) of the main text. The assessment of f_c also required the knowledge of the atmospheric concentration, C_a, the measured value of which was close to 400 ppm throughout the whole experiment (see Methods). The results are summarized in Supplementary Table 2. For the maximum observed discharge (i.e. $Q = 2.11$ l/s), f_{c_r} was equal 0.09 corresponding to an excess mass removed of 8.3%. For intermediate discharge levels (i.e., $Q = 0.73$ l/s), the maximum estimated f_{c_r} was equal 0.32 with a percentage of initial mass evaded equal 27.7%. A possible explanation of the decrease of f_{c_r} for the highest discharges lies in the dependence of the area–to–volume ratio (SA/V) on discharge. In aquatic systems, high SA/V increases the gas exchange with the atmosphere. In the reference segment, the values of SA/V in the representative segment were experimentally estimated under the assumption of rectangular cross-section and rectangular air-water surface (see Supplementary Table 3). The exchange area, SA, was estimated as the product between the average top width or wetted width, W, and the length of the representative segment, ℓ_r. The volume V is the product between the SA and the mean water depth, H. The ratio between this two quantities decreases with Q, thereby indicating a decrease of the efficiency of gas exchange in the high discharge configuration. The relatively low value of the continuous damping factor for the smallest value of discharge, instead, was likely affected by the low mean velocity of the flow field under these flow conditions. Low water velocities, in fact, are know to originate less turbulence and reduced CO_2 outgassing from rivers.

We have also compared the values of f_c observed in this study with f_c estimates as derived from previously published data. In particular, we calculated f_c for a reference length of 1 m from the data reported in Ulseth et al.[3] as $f_c(1) = (k/H\,L)\,\tau$, where k was estimated from the corresponding value of k_{600} reported in that paper assuming a reference temperature of $12\,°C$. The frequency distribution of $f_c(1)$ derived for all the streams analyzed in Ulseth et al.[3] is shown in Supplementary Figure 11, jointly with a comparison with the values of $f_c(1) = f_{c_r}/\ell_r$ estimated in this study. $f_c(1)$ values obtained here were thus in line with the values of $f_c(1)$ derived from previously published data, suggesting the robustness of our estimates of the outgassing rate from the reference segment.

Starting from the values of f_{c_r} we also computed, for the different discharge levels investigated in this study, the corresponding exchange rates $K = f_{c_r}/\tau_{c_r}$ and the mass transfer rates $k = K\,H$ (where τ_{c_r} and H are the water travel time and the stage, respectively). Then, k values were transformed into standardized mass exchange rates k_{600} through a temperature-dependent Schmidt scaling, so as to facilitate the comparison of our measurements with previous studies. we also computed the fluxes of CO_2 into the atmosphere (F^* [M/T]) as $F^* = Q(C(0) - C(\ell_r))$, and the corresponding areal fluxes (F [M/T/L^2]) as $F = F^*/(L_r\,W)$. The results of the above calculations are reported in Supplementary Table 4.

Supplementary Table 2: Mean (μ) and standard deviation (SD) of measured CO_2 concentrations in the upstream (0) and downstream (ℓ_r) sections of the representative segment. In the last row the estimated damping factors, f_{c_r}, with $C_a = 400\ ppm$.

Date	*Sept.*10, 2021		*Sept.*21, 2021		*Oct.*7, 2021		*Oct.*20, 2021	
Q (l/s)	0.19		0.37		2.11		0.73	
	0	ℓ_r	0	ℓ_r	0	ℓ_r	0	ℓ_r
μ (ppm)	1243	1104	1051	879	966	919	1115	917
SD (ppm)	12.47	16.49	15.31	10.64	12.83	14.15	16.80	8.87
f_{c_r}	0.181		0.305		0.089		0.321	

Supplementary Table 3: Estimated surface area–to–volume ratio, SA/V, for measured discharges. The exchange area, SA, is the product between the average top width or wetted width, W (m), and the length of the representative segment, ℓ_r (m). The volume V is the product between the SA and the mean water depth, H (m).

	Q = 0.19 l/s		Q = 0.37 l/s		Q = 0.73 l/s		Q = 2.11 l/s	
L	W	H	W	H	W	H	W	H
13	0.08	0.032	0.12	0.035	0.16	0.042	0.3	0.048
SA (m^2)	1.04		1.56		2.08		3.90	
V (m^3)	0.03		0.05		0.09		0.19	
SA/V (m^{-1})	31.25		28.36		23.81		20.83	

Supplementary Table 4: CO_2 fluxes and k_{600} from the reference segment. The Table reports for each discharge tested, the upstream (C_u) and downstream (C_d) concentrations, the discharge Q, the standardized mass transfer rate k_{600}, the areal (F) and total (F^*) CO_2 flux released to the atmosphere. Mean values and standard deviations of C_u, C_d, F and F^* are also shown in the last two lines of the table.

	C_u [ppm]	C_d [ppm]	Q [l/s]	k_{600} [m/d]	F [$gC\ m^{-2}\ d^{-1}$]	F^* [$gC\ d^{-1}$]
	1243	1104	0.19	3.08	2.66	1.01
	1050	879	0.37	7.55	4.70	2.70
	966	897	2.11	8.42	4.68	6.68
	1113	917	0.73	12.58	9.34	7.10
μ	1093	949			5.34	4.36
SD	117	104			2.82	2.99

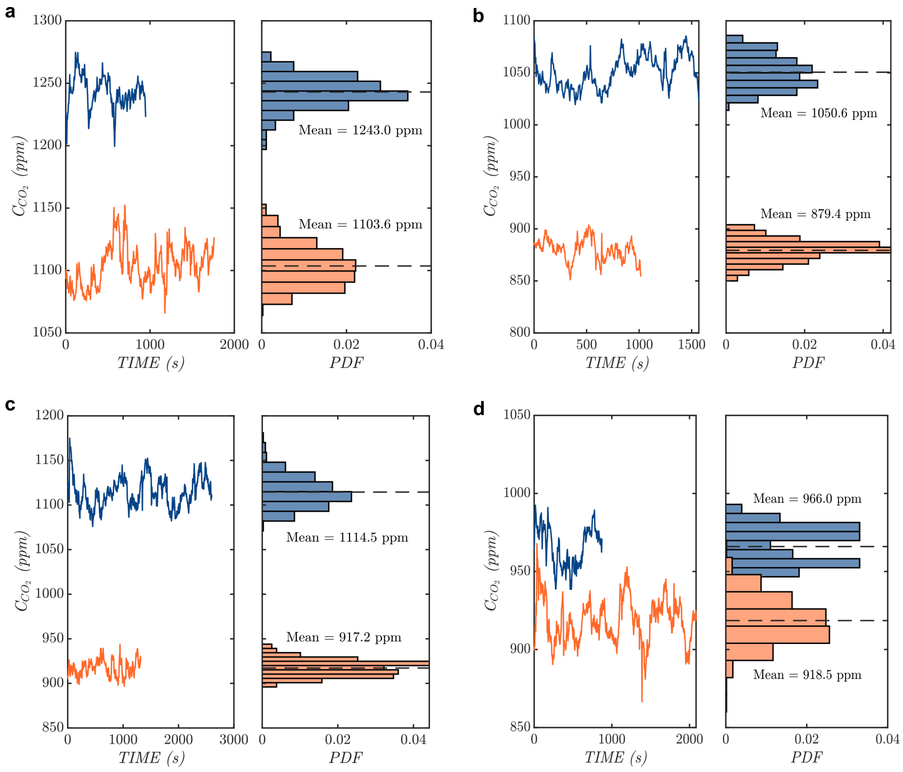

Supplementary Fig. 10: Time series of upstream (blue) and downstream (orange) CO_2 concentrations in the representative segment with PDFs and mean values on the right. Measured discharge was on (**a**) 0.19 l/s, on (**b**) 0.37 l/s, on (**c**) 0.73 l/s and on (**d**) 2.11 l/s.

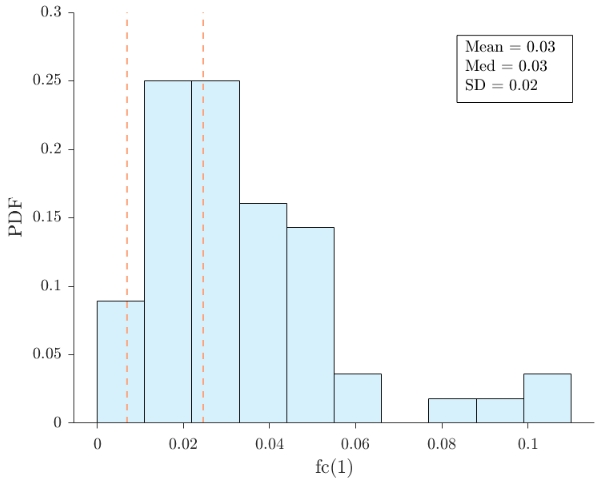

Supplementary Fig. 11: Frequency distribution of f_c calculated for a reference length of 1 m, derived for all the streams analyzed in Ulseth et al.[3]. The range of values of values of f_{c_r} estimated in this study is also shown.

1.5.4 CO_2 concentrations, CO_2 fluxes and damping factors for the steps

Carbon dioxide concentrations were collected from September to November 2021 in the upstream and downstream sections of 19 different steps of the Valfredda river. The time series of $\mathrm{CO_2}$ concentrations are shown in Supplementary Figure 10, jointly with the corresponding pdfs. We only retained those measurement pairs in which the upstream-downstream difference was larger than the sum of the two standard deviations of each timeseries. The discarded pairs were 3 out of 23, and corresponded to the cases with the lowest CO_2 concentrations and the lowest values of f. The assessment of the damping factors also required the knowledge of the atmospheric concentration, C_a, the measured value of which was close to $400ppm$ throughout the whole experiment (see Methods).

The damping factors of the steps were calculated from paired upstream / downstream concentration measurements according to Equation (10) in the main text. The results are summarized in Supplementary Table 5. The damping factor f_{s_i} was found to be independent on the discharge Q. This result was formally proven using the Chi-Square Test of Independence. The application of the test to our dataset allowed us to conclude that there is not enough evidence to suggest an association between Q and f_{s_i}. As the obtained p-value was greater than our chosen significance level ($\alpha = 0.05$), the null hypothesis of statistical dependence between f_{si} and Q was rejected ($\chi^2(6) > 3.306,\ p = 0.770$).

Starting from the the observed values of CO_2 concentrations, the total fluxes of CO_2 released into the atmosphere through the steps, F^*, were then estimated

as $F^* = Q(C_u - C_d)$. The results of the calculations are reported in Supplementary Table 6. Interestingly, the mean value of F^* was similar to the mean value of F^* obtained for the reference segment, indicating a similar contribution to the outgassing.

Supplementary Table 5: Mean (μ) and standard deviation (SD) of measured CO_2 concentrations upstream (u) and downstream (d) the steps of drop height Δh_i, referring to Figure 12 from (**a**) to (**l**). In the grey rows the estimated damping factors, $f_{s_i}(\Delta h_i)$, with $C_a = 400$ ppm.

Date	$Sept.10, 2021$					
Δh_i (cm)	26.5		57		83	
	u	d	u	d	u	d
μ (ppm)	1297	1221	1284	1110	1287	1116
SD (ppm)	15.87	16.03	29.20	13.78	14.33	16.51
$f_{s_i}(\Delta h_i)$	0.089		0.218		0.269	
Date	$Sept.21, 2021$					
Δh_i (cm)	26		52		63	
	u	d	u	d	u	d
μ (ppm)	1100	1061	1130	1027	1156	1031
SD (ppm)	14.90	16.06	14.13	17.61	13.40	19.05
$f_{s_i}(\Delta h_i)$	0.056		0.152		0.181	
Date	$Sept.30, 2021$					
Δh_i (cm)	32		53		25	
	u	d	u	d	u	d
μ (ppm)	1149	1097	1228	1120	1236	1184
SD (ppm)	14.30	16.07	22.30	9.03	15.22	12.04
$f_{s_i}(\Delta h_i)$	0.071		0.140		0.064	

Supplementary Table 5: Mean (μ) and standard deviation (SD) of measured CO_2 concentrations upstream (u) and downstream (d) the steps of drop height Δh_i, referring to Figure 12 from (**m**) to (**s**). In the grey rows the estimated damping factors, $f_{s_i}(\Delta h_i)$, with $C_a = 400$ ppm.

Date	$Oct.07, 2021$					
Δh_i (cm)	20		45		73	
	u	d	u	d	u	d
μ (ppm)	1000	966	1062	963	1062	936
SD (ppm)	11.42	12.83	16.46	13.44	16.46	15.17
$f_{s_i}(\Delta h_i)$	0.058		0.162		0.211	
Date	$Oct.11, 2021$					
Δh_i (cm)	20		49		58	
	u	d	u	d	u	d
μ (ppm)	1070	1049	1992	1807	1140	1042
SD (ppm)	8.72	7.41	15.42	18.81	12.53	16.07
$f_{s_i}(\Delta h_i)$	0.032		0.124		0.142	
Date	$Oct.28, 2021$					
Δh_i (cm)	23		40		43	
	u	d	u	d	u	d
μ (ppm)	984	932	1040	969	572	553
SD (ppm)	22.22	10.32	21.52	15.96	10.70	9.69
$f_{s_i}(\Delta h_i)$	0.092		0.117		0.127	

Date	$Nov.12, 2021$	
Δh_i (cm)	25	
	u	d
μ (ppm)	823	799
SD (ppm)	12.78	11.06
$f_{s_i}(\Delta h_i)$	0.059	

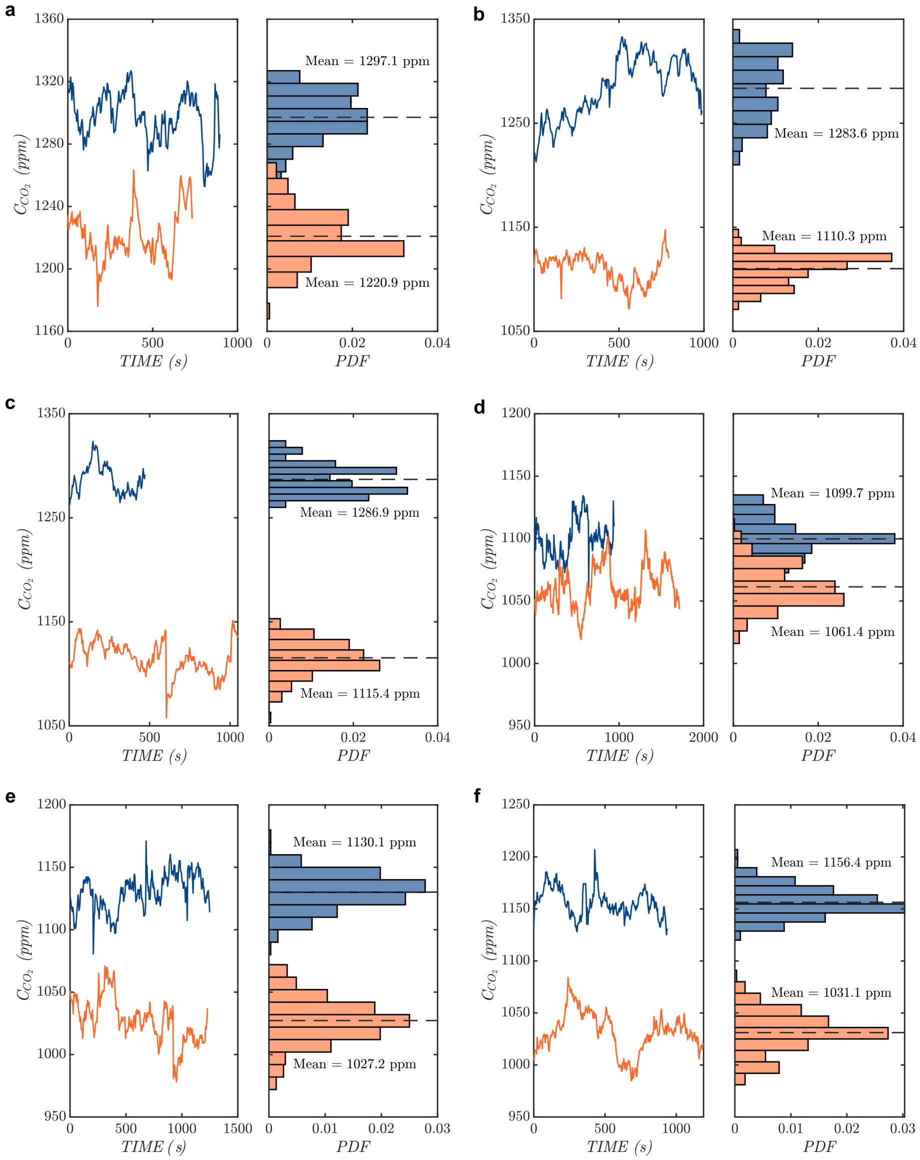

Supplementary Fig. 12: Time series of upstream (blue) and downstream (orange) CO_2 concentrations in artificial steps. PDFs and mean values are shown on the right. (**a**), (**b**) and (**c**) were collected on 10th September 2021, (**d**), (**e**) and (**f**) on 21th September 2021.

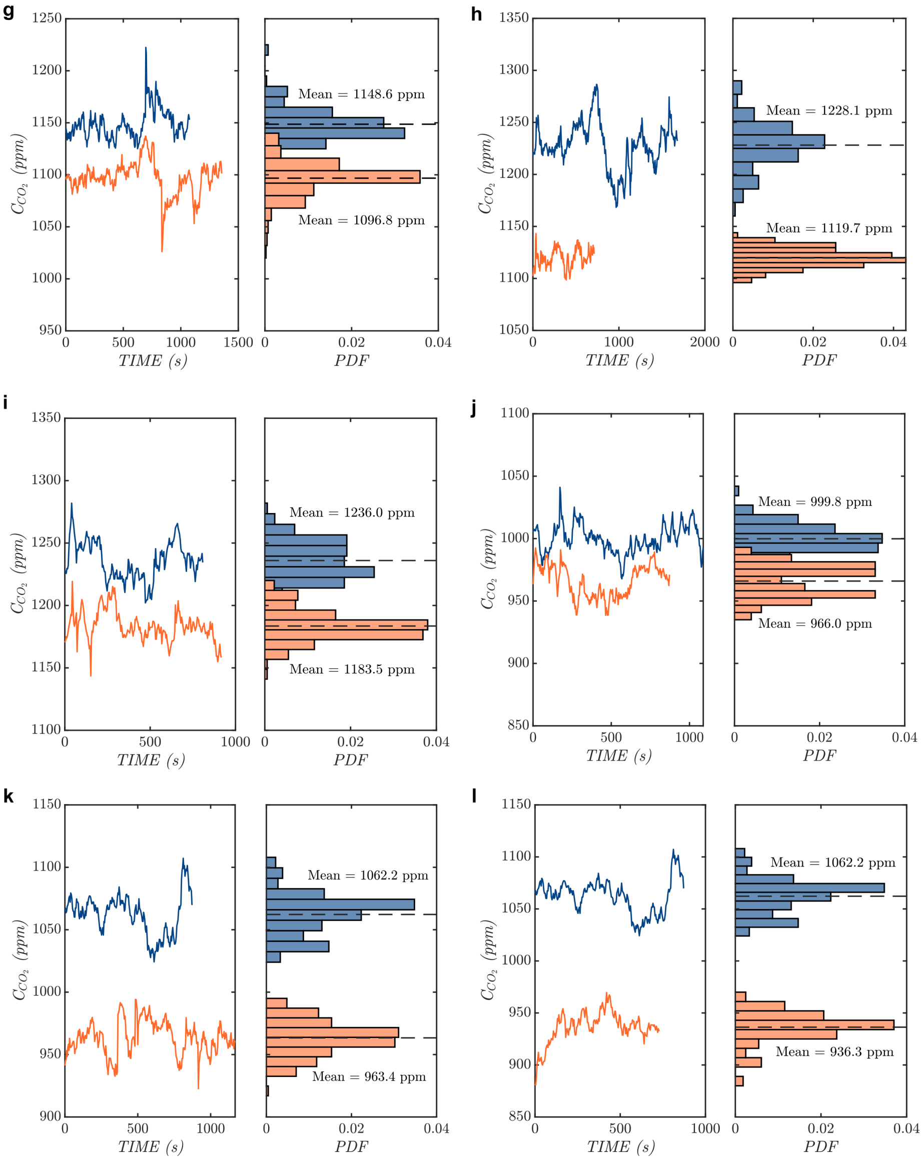

Supplementary Fig. 12: Time series of upstream (blue) and downstream (orange) CO_2 concentrations in artificial steps and on (**g**) in natural step in covered condition. PDFs and mean values are shown on the right. (**g**), (**h**) and (**i**) were collected on 30th September 2021, (**j**), (**k**) and (**l**) on 7th October 2021.

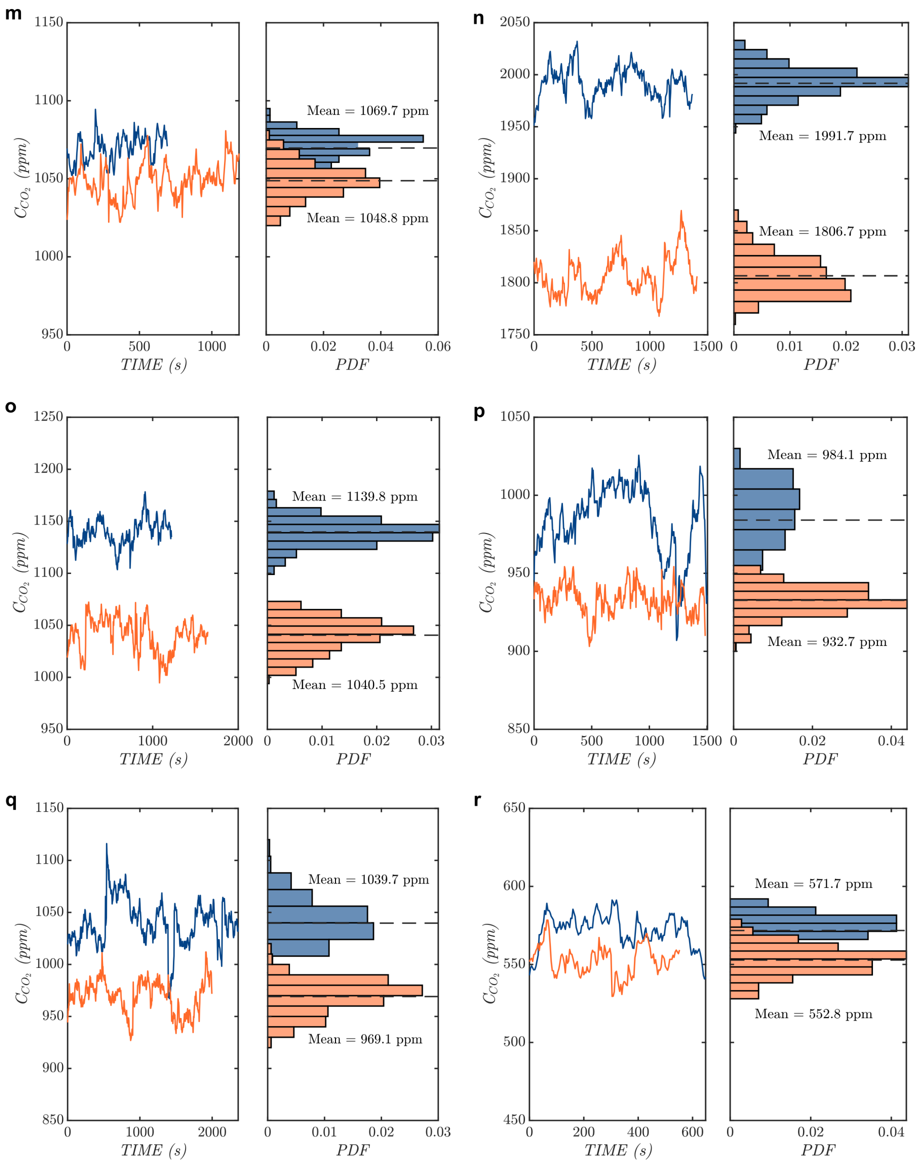

Supplementary Fig. 12: Time series of upstream (blue) and downstream (orange) CO_2 concentrations with PDFs and mean values on the right. (**m**), (**n**) and (**o**) referred to natural step in covered condition collected on 11th October 2021; (**p**), (**q**) and (**r**) referred to natural step in uncovered condition collected on 28th October 2021.

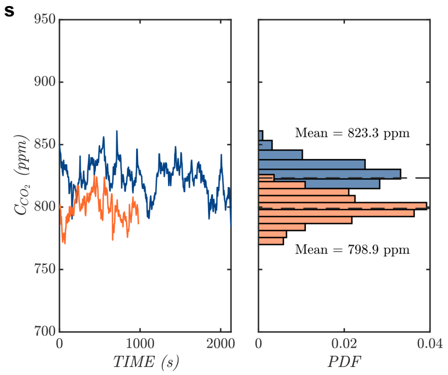

Supplementary Fig. 12: Time series of upstream (blue) and downstream (orange) CO_2 concentrations with PDFs and mean values on the right. (**s**) referred to natural step in uncovered condition collected on 12th November 2021.

1.6 Upscaling procedure

In this paper, different scenarios were considered, in which the damping factor of the reference segment was upscaled via Eq. (9) of the main text with specific reference to three different reaches: i) the reach A, which represents the whole accessible part of the tributary considered in this study; ii) the virtual reach A^*, which represents a virtual reach in which the continuous segments have a mean slope equal to that of the reference segment and the total elevation drop of the reach is the same as that experienced by the flow in reach A; iii) the reach B, which is the upper portion of reach A and is characterized by the fact that its segments have a mean slope equal to that of the continuous segments. The reach A has a length of 1060 m (light blue in Figure 1**a**). The drainage area at the downstream section of A is about 0.29 km^2 with a total mean slope of $0.142\,m/m$. The mean slope of the continuous part of A is smaller than the slope of the representative segment ($0.108\,m/m$). Therefore, the use of f_c calculated herein to describe continuous outgassing processes in the whole reach would inevitably underestimate the dominance ratio. For this reason, the computation of r was also performed considering a virtual reach, A^*, in which all the underlying segments are characterized by the same slope of the representative segment. The equivalent length of this idea reach was calculated through a proportion between vertical gradient and length in the representative segment and in the continuous part of A. The stretch B, instead, is characterized by continuous segments with a mean slope very similar to the slope of the 13 m representative segment. The outlet of B has an altitude of 1820 m $a.s.l$, 543 m downstream of the source, leading to a total slope of this reach (segments plus steps) of $0.168\,m/m$.
For the three reaches used in the upscaling (A, B, and A^*), the values of r shown in Figure 4**c** of the main text were calculated from the spatial frequency

Supplementary Table 6: CO_2 fluxes from the steps. The Table reports for each step, the upstream (C_u) and downstream (C_d) concentrations, the discharge Q, the step height Δh_i and the total CO_2 flux released to the atmosphere. Mean values and standard deviations of C_u, C_d and F^* are also shown in the last two lines of the table. In the computation of these statistics the step belonging to the main Valfredda river, which has a discharge much higher than all the other steps and is reported with an asterisk in the table, has been disregarded.

	C_u $[ppm]$	C_d $[ppm]$	Q $[l/s]$	$\Delta\, h_i$ $[cm]$	F^* $[gC\ d^{-1}]$
	1297	1221	0.20	0.27	0.58
	1284	1110	0.20	0.57	1.30
	1287	1116	0.20	0.83	1.32
	1100	1061	0.37	0.26	0.60
	1130	1027	0.37	0.52	1.62
	1156	1031	0.37	0.63	1.97
	1236	1184	0.74	0.25	1.92
	1228	1120	0.74	0.53	3.97
	1000	966	2.11	0.20	3.29
	1062	963	2.11	0.45	9.56
	1062	936	2.11	0.73	12.19
	1992	1807	1.90	0.49	15.40
	1140	1042	0.71	0.58	3.04
	984	932	0.35	0.23	0.79
	1040	969	1.05	0.40	3.26
	823	799	0.83	0.25	0.88
	1070	1049	0.55	0.20	0.49
	1149	1097	0.25	0.32	0.58
	572*	553*	108*	0.43*	89.86*
μ	1169	1079			3.48
SD	239	207			4.36

Supplementary Table 7: Summary information of the morphological survey; N is the number of the mapped steps, Δh_s is the sum of the collected step drop heights, H_c is the vertical gradient of the continuous part (i.e. the total elevation difference minus Δh_{TOT}), L_c is the reach length excluding 10 cm for each step - for A^* is the equivalent length - and i_c is the mean slope of the segments included in the reach.

	N	Δh_{TOT} $[m]$	H_c $[m]$	L_c $[m]$	i_c $[m/m]$
ℓ_r			1.4	13	0.108
A	271	67.7	83.4	1033	0.081
A^*	271	67.7	83.4	769	0.108
B	130	37.4	53.7	530	0.101

of steps with a given drop height (Δh) included in the reach (Supplementary Figure 13) and using a linear empirical law to link f_{s_i} to Δh_i (see Figure 3**a**). In particular, r was expressed as:

$$r = L \sum_{\Delta h} \frac{\lambda_{\Delta h} f_s(\Delta h)}{f_c(L)} \ , \tag{12}$$

where $\lambda_{\Delta h}$ is the spatial frequency of steps with a given height in the focus reach, $f_c(L)$ the damping factor of the segments in the reach, L their total length and $f_s(\Delta h)$ is given by the following linear regression:

$$f_s(\Delta h) = 0.3\,\Delta h \ , \tag{13}$$

as suggested by Figure 3a of the main text, for which the goodness of fit is $R^2 = 0.978$ (Δh is expressed in m). As per the calculation of $\lambda_{\Delta h}$, we exploited a morphological survey of the reach during which we collected data about all the drops in the riverbed higher than $10\,cm$. The surveyed step heights were divided into classes and the corresponding spatial frequency was defined by $N_{\Delta h}$, the number of steps with a height range around Δh in the considered reach:

$$\lambda_{\Delta h} = \frac{N_{\Delta h}}{L} \ . \tag{14}$$

In reach A, the frequency of lower height steps was greater than frequency of high drops, as emerging from the monotonically decreasing behaviour of $\lambda_{\Delta h}$ shown in Supplementary Figure 13.

Supplementary Table 8 provides information about the contribution to the total f_s provided by different step classes. Similar results were also gathered for reach A^* and B.

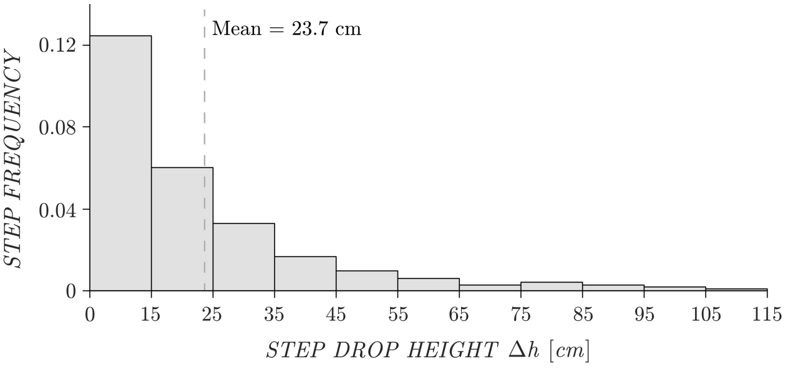

Supplementary Fig. 13: Steps spatial frequency distribution in reach A, corresponding to 271 mapped steps in 1.06 *km* with an average drop height equal 23.7 *cm*.

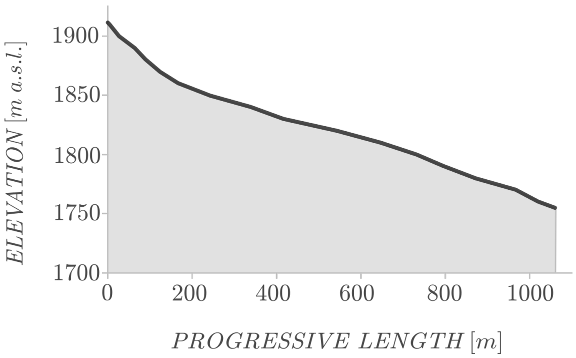

Supplementary Fig. 14: Longitudinal profile of reach A from 1911 to 1760 *m a.s.l.* for a total length of 1.06 *km*.

Supplementary Table 8: Step drop height classes and corresponding contribution to f_s in reach A.

Step classes [cm]	Contribution to f_s
0 - 15	25.6 %
15 - 25	20.5 %
25 - 35	15.8 %
35 - 45	10.1 %
45 - 55	7.3 %
55 - 65	5.2 %
65 - 75	3.0 %
75 - 85	4.5 %
85 - 95	3.8 %
95 - 105	2.8 %
105 - 115	1.5 %

1.7 Dependence of the outgassing on the internal structure of a reach

If steps are observed within a reach, the damping factor and the mass evaded do not depend only on the mean properties of the reach (e.g. the mean slope). Rather, the actual outgassing also depends on the internal structure of the reach. To provide an example of this important result, we have compared the outgassing observed in the reference segment of length ℓ_r to that produced by an ideal reach with the same mean slope and length of the reference segment, but with a different internal structures (two horizontal segments and two steps, as shown in Supplementary Figure 15). This ideal reach, made up of 2 steps of drop height $\Delta h_i = 0.7\,m$ and two horizontal segments (shown on the right) has the same mean slope and elevation drop ($1.4\,m$) of the representative segment with length $\ell_r = 13\,m$ (represented on the left). The excess mass removed within the representative segment is between 8.3 % ($f_{c_r} = 0.09$) and 27.7 % ($f_{c_r} = 0.32$), depending on the underlying discharge rate. Assuming no outgassing in the horizontal segments, a conservative estimate of the damping factor in this ideal two-step-reach is obtained, which is equal to 0.42. The corresponding evaded mass would be approximately equal to 33%, which is significantly larger than that outgassed by the reference segment.

1.8 Streamflow regime

Daily streamflow temporal dynamics of the representative segment were modelled using Eq. (10) in the main text (Figure 4**b**). The relevant contributing area, A, was 0.11 km^2. The calibrated model parameters, obtained minimizing the mean squared error between the model and measured discharges (Supplementary Table 1), were the following: $\phi = 16.7$ *mm* and $k = 0.062$ *1/d*.

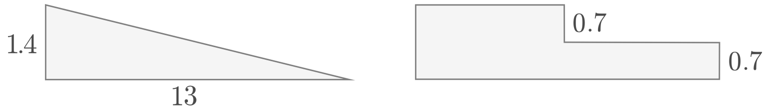

Supplementary Fig. 15: On the left, the representative segment with length $\ell_r = 13\,m$ and elevation drop $1.4\,m$. On the right, the same mean slope and elevation drop ($1.4\,m$) is given by the combination of two steps of drop height $\Delta h_i = 0.7\,m$ and two horizontal segments.

Supplementary References

1. Hirt, C. W. & Nichols, B. D. Volume of fluid (VOF) method for the dynamics of free boundaries. *Journal of computational physics* **39,** 201–225 (1981).
2. Moog, D. B. & Jirka, G. H. Stream reaeration in non uniform flow: macroroughness enhancement. *Journal of Hydraulic Engineering* **125(1),** 11–16 (1999).
3. Ulseth, A. J. *et al.* Distinct air–water gas exchange regimes in low-and high-energy streams. *Nature Geoscience* **12,** 259–263 (2019).
